# S100B dysregulation during brain development affects synaptic SHANK protein networks via alteration of zinc homeostasis

**DOI:** 10.1038/s41398-021-01694-z

**Published:** 2021-11-05

**Authors:** Eleonora Daini, Simone Hagmeyer, Chiara A. De Benedictis, Joana S. Cristóvão, Martina Bodria, Aisling M. Ross, Andrea Raab, Tobias M. Boeckers, Joerg Feldmann, Cláudio M. Gomes, Michele Zoli, Antonietta Vilella, Andreas M. Grabrucker

**Affiliations:** 1 Department of Biomedical, Metabolic and Neural Sciences, Center for Neuroscience and Neurotechnology, University of Modena and Reggio Emilia, Modena, Italy; 2 Cellular Neurobiology and Neuro-Nanotechnology lab, Department of Biological Sciences, University of Limerick, Limerick, Ireland; 3 Bernal Institute, University of Limerick, Limerick, Ireland; 4 Biosystems and Integrative Sciences Institute Faculdade de Ciências, Universidade de Lisboa, Universidade de Lisboa, Lisbon, Portugal; 5 Departamento de Química e Bioquímica, Faculdade de Ciências, Universidade de Lisboa, Lisboa, Portugal; 6 Health Research Institute (HRI), University of Limerick, Limerick, Ireland; 7 Trace Element Speciation Laboratory (TESLA), Department of Chemistry, University of Aberdeen, Aberdeen, Scotland UK; 8 Trace Element Speciation Laboratory (TESLA), Institute for Chemistry, University of Graz, Graz, Austria; 9 Institute for Anatomy and Cell Biology, Ulm University, Ulm, Germany; 10 DZNE, Ulm Site, Ulm, Germany

**Keywords:** Molecular neuroscience, Physiology

## Abstract

Autism Spectrum Disorders (ASD) are caused by a combination of genetic predisposition and nongenetic factors. Among the nongenetic factors, maternal immune system activation and zinc deficiency have been proposed. Intriguingly, as a genetic factor, copy-number variations in *S100B*, a pro-inflammatory damage-associated molecular pattern (DAMP), have been associated with ASD, and increased serum S100B has been found in ASD. Interestingly, it has been shown that increased S100B levels affect zinc homeostasis in vitro. Thus, here, we investigated the influence of increased S100B levels in vitro and in vivo during pregnancy in mice regarding zinc availability, the zinc-sensitive SHANK protein networks associated with ASD, and behavioral outcomes. We observed that S100B affects the synaptic SHANK2 and SHANK3 levels in a zinc-dependent manner, especially early in neuronal development. Animals exposed to high S100B levels in utero similarly show reduced levels of free zinc and SHANK2 in the brain. On the behavioral level, these mice display hyperactivity, increased stereotypic and abnormal social behaviors, and cognitive impairment. Pro-inflammatory factors and zinc-signaling alterations converge on the synaptic level revealing a common pathomechanism that may mechanistically explain a large share of ASD cases.

## Introduction

Autism Spectrum Disorders (ASD) are a group of neurological disorders considered to manifest from a synaptic dysfunction or synaptopathy [1]. Genetic factors might be largely causative for the development of ASD and alone or in combination with specific environmental risk factors trigger the pathology. Among the nongenetic factors, a potential role of maternal inflammation and immune system alteration, and prenatal zinc deficiency have been proposed [2, 3]. So far, it is unclear how the plethora of identified ASD candidate genes and environmental factors all result in the same behavioral abnormalities characteristic of ASD. However, it can be speculated that the different factors converge on the same cellular biological pathway that lies at the core of ASD pathology. In this study, we elucidate how pro-inflammatory processes are linked to zinc homeostasis and how both inflammatory processes and zinc signaling ultimately affect synaptic plasticity via effects on known ASD candidate genes and their encoded proteins.

Damage-associated molecular patterns (DAMPs) such as neural S100 proteins are increased during inflammatory processes activating NF-κB [4]. S100B was found increased in brain disorders associated with inflammation, such as Alzheimer’s disease, motor neuron disease, and traumatic brain injury [5–10]. However, copy-number variations of *S100B* are also associated with the etiology of ASD [11]. Autistic children also showed significantly higher serum S100B levels than healthy controls, and S100B protein levels were significantly correlated to the severity of autism [12].

The expression level of S100B proteins increases in response to factors upregulated during infections such as TNFα, IL-1β, IL-6, and IL-8 [13–15], also reported to be increased in prenatal infection models [16]. S100 proteins are a family of low molecular weight (MW), regulatory, zinc- and calcium-binding proteins [4]. A previous study shows that increased levels of S100B sequester zinc ions in vitro [17]. High (μM) levels of S100B thereby significantly affect intracellular zinc levels of nearby neurons. While acutely elevated levels of S100B may act neuro-protective by buffering zinc and thereby regulating calcium-signaling, which may ultimately reduce excitotoxicity [17], chronically elevated levels of S100B may impair the physiological role of zinc in synapse plasticity and function due to increased zinc sequestration.

Zinc is a structural and regulatory component of many proteins but also acts as a neurotransmitter/neuromodulator and signaling ion in the brain [18–22]. On a cellular level, zinc signaling was shown to play a role in neuronal differentiation and synapse formation, maturation, and plasticity [23]. Especially synaptogenesis and function of excitatory glutamatergic terminals are influenced by zinc signaling that regulates, among others, the dynamics of SHANK2 and SHANK3 scaffold proteins at the post-synaptic density (PSD) [24–26]. Given that *SHANK2* and *SHANK3* are major autism candidate-genes [27–33], their regulation via zinc may provide a link between genetic and non-genetic risk factors in ASD.

Here, we hypothesize that hypozincemia induced by increased levels of S100B during brain development will affect synapse function and maturation, possibly via the autism-associated SHANK protein complex at the PSD [24, 34]. Impaired zinc signaling involved in both inflammatory processes and synaptic function might ultimately trigger lasting pathological processes related to ASD during brain development [26, 35]. Thus, we investigated the impact of altered S100B protein levels on synapse formation and proteins of the SHANK family in vitro and in vivo and assessed the influence of prenatal exposure to elevated S100B levels on the behavior of mice.

## Results

### S100B modulates synaptic SHANK2 and SHANK3 levels in a zinc-dependent manner

S100B is primarily expressed in the central nervous system by astrocytes releasing S100B into the brain parenchyma. It accounts for 0.5% of the brain’s soluble proteins [36–38]. We confirmed that S100B is present in all major brain regions (Supplementary Fig. S1a, b). Analysis of mouse brain lysates shows the expression of S100B in the cortex, hippocampus (HIP), striatum (STR), and cerebellum both at mRNA (Supplementary Fig. S1a) and protein (Supplementary Fig. S1b) level. Endogenous S100B proteins are found in soluble S1 and P2 (crude membrane) protein fractions (Supplementary Fig. S1b). Immunocytochemistry performed on hippocampal neurons shows that after increasing S100B levels, S100B added to the cell culture medium co-localizes with a synaptic marker protein (Supplementary Fig. S1c). Therefore, synapses may be specifically modulated through high levels of S100B.

**Fig. 1:**
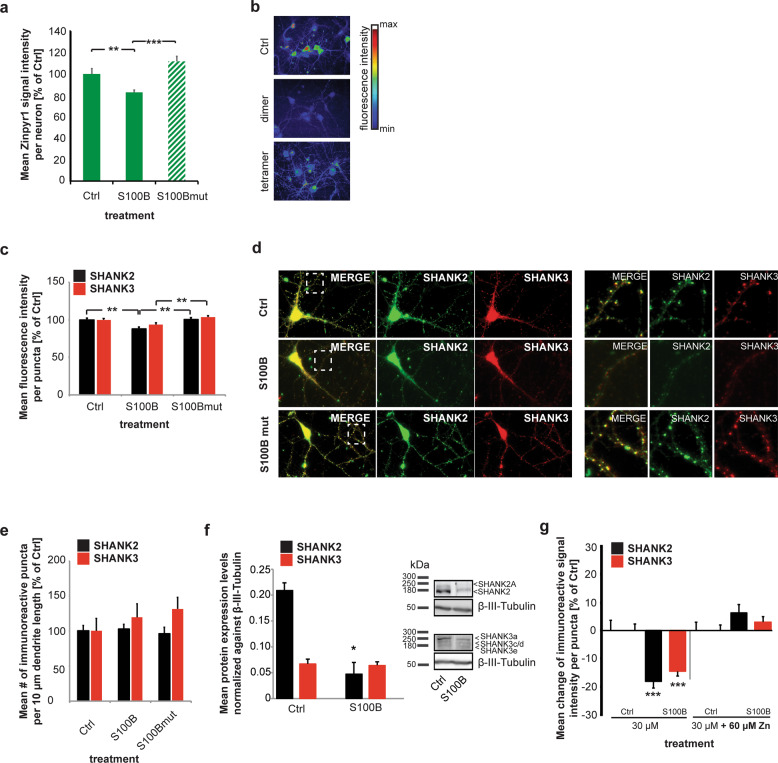
Increased levels of S100B sequester zinc ions. **a** Treatment of hippocampal cultures with 30 μM S100B or S100Bmut for 24 h at DIV10. The mean intracellular signal intensity of Zinpyr1 fluorescence is shown. Treatment of hippocampal cultures with S100B significantly reduces the free intracellular Zn^2+^ concentration assessed by the analysis of 35 cells per condition. No significant decrease in intracellular Zinpyr1 fluorescence can be observed in cultures treated with S100Bmut (one-way ANOVA, *F*_2,77_ = 8.86; *p* < 0.0001; Post-hoc analysis: control vs. S100B, *p* = 0.0037; control vs. S100Bmut, *p* = 0.2469; S100B vs. S100Bmut, *p* < 0.0001). **b** Exemplary images showing signal intensities of Zinpyr1 in a color-coded manner. **c**–**g** Increased levels of S100B lead to a decrease in zinc-dependent SHANK proteins at the synapse. **c** Treatment of hippocampal cultures with 30 μM S100B for 24 h. The signal intensity of SHANK2 is significantly reduced 24 h after exposure to S100B. Similarly, the signal intensity of SHANK3 puncta shows a trend toward a reduction. No significant reduction of SHANK2 and SHANK3 was observed 24 h after applying S100Bmut. For all analyses, 8–10 cells per condition were used (one-way ANOVA: SHANK2: *F*_2,27_ = 502.009, *p* = 0.001; Post-hoc analysis: Control vs. S100B *p* = 0.0013, S100B vs. S100Bmut *p* = 0.0029; Shank3: *F*_2,27_ = 278.257, *p* = 0.005; Post-hoc analysis: Control vs. S100B *p* = 0.497, S100B vs. S100Bmut *p* = 0.0037) (*n* = 10 cells per condition). **d** Exemplary images showing anti-SHANK2 and SHANK3 staining of hippocampal neurons 24 h after treatment with S100B and S100Bmut. **e** The mean number of immunoreactive signals per dendrite length was evaluated. No significant alterations were detected. **f** Using Western blot experiments (from three different preparations), a significant decrease of SHANK2 protein levels in the crude membrane fraction can be seen (*t*-test, *p* = 0.0265). **g** Cells were again treated with S100B for 24 h at DIV8. A significant reduction of SHANK2 and SHANK3 proteins is visible. This reduction was abolished in case cells were treated with 30 μM S100B that was saturated with 60 μM ZnCl_2_ for 1 h on ice before application (one-way ANOVA: SHANK2: *F*_3,36_ = 12.2454, *p* < 0.001; Post-hoc analysis: Control vs. S100B *p* = 0.001, S100B vs. saturated S100B *p* = 0.001; SHANK3: *F*_3,36_ = 15.9105, *p* = 0.005; Post-hoc analysis: Control vs. S100B *p* = 0.001, S100B vs. saturated S100Bmut *p* = 0.001, *p* = 0.0037) (*n* = 10 cells per condition).

Given that increased levels of S100B have been reported in ASD [12], in the first set of experiments, we treated primary hippocampal neurons from rats with 30 μM S100B for 24 h. This concentration has been previously reported non-toxic to neurons and significantly affects cellular zinc homeostasis [17]. The non-toxicity of 30 μM S100B was confirmed in this study (Supplementary Fig. S2a). We could verify the previously reported decrease in intracellular zinc observed by the exposure of neurons to S100B using Zinpyr1 (Fig. 1a, b). Zinpyr1 is a cell-permeable, fluorescein-based probe that selectively detects free and weakly bound zinc. A significantly reduced intracellular zinc concentration assessed by the analysis of Zinpyr1 signal intensity was measured (Fig. 1a). This decrease was absent using a zinc-binding mutant S100B protein (S100Bmut), with Serine replacements of the amino acids involved in zinc binding (His15, His25, Cys84, and His85) [38, 39]. Mutant S100B has been characterized previously [17], and impaired zinc-binding with intact conformational stability was confirmed. The exposure of rat primary hippocampal neurons to 30 μM mutated S100B for 24 h did not significantly reduce intracellular zinc levels (Fig. 1a).

Next, we wanted to analyze whether the decrease in intracellular zinc levels leads to destabilization of SHANK2 and SHANK3 complexes at the PSD of excitatory synapses as previously reported [24–26, 40]. Using immunocytochemistry, we evaluated the signal intensity of the two zinc-dependent Shank family members SHANK2 and SHANK3 at synapses (Fig. 1c–e). Specificity of the antibodies for SHANK2 or SHANK3 has been confirmed previously [26]. We could detect a significant reduction of synaptic SHANK2 levels 24 h after exposure to S100B (Fig. 1c, d) and a trend toward a decrease of SHANK3. The effects of S100B and S100Bmut were significantly different regarding both SHANK2 and SHANK3. Mutant non-zinc-binding S100B did not significantly reduce SHANK2 or SHANK3 levels (Fig. 1c). The overall number of immunoreactive signals per dendrite length (Fig. 1e) and dendritic arborization (Supplementary Fig. S2b, c) were unaffected within 24 h of treatment. Further, no effects were detected at inhibitory synapses that were assessed using the marker protein Gephyrin (Supplementary Fig. S2d). All these changes were also confirmed by using different approaches and, specifically, the decrease of SHANK2 protein levels in crude membrane fractions 24 h after exposure to S100B was also demonstrated by Western blot (WB) analysis (Fig. 1f).

We repeated the experiment using S100B pre-incubated (saturated) with zinc before the in vitro application (Fig. 1g). Non-saturated S100B, again, caused a significant decrease in synaptic SHANK2 signal intensity and also affected SHANK3 levels significantly in this experiment. In contrast, zinc-saturated S100B did not significantly alter SHANK2 and SHANK3 signal intensities at synapses (Fig. 1g). Altered zinc levels through the application of S100B did not change the transcriptional levels of *Shank2* or *Shank3* genes (Supplementary Fig. S2e).

**Fig. 2:**
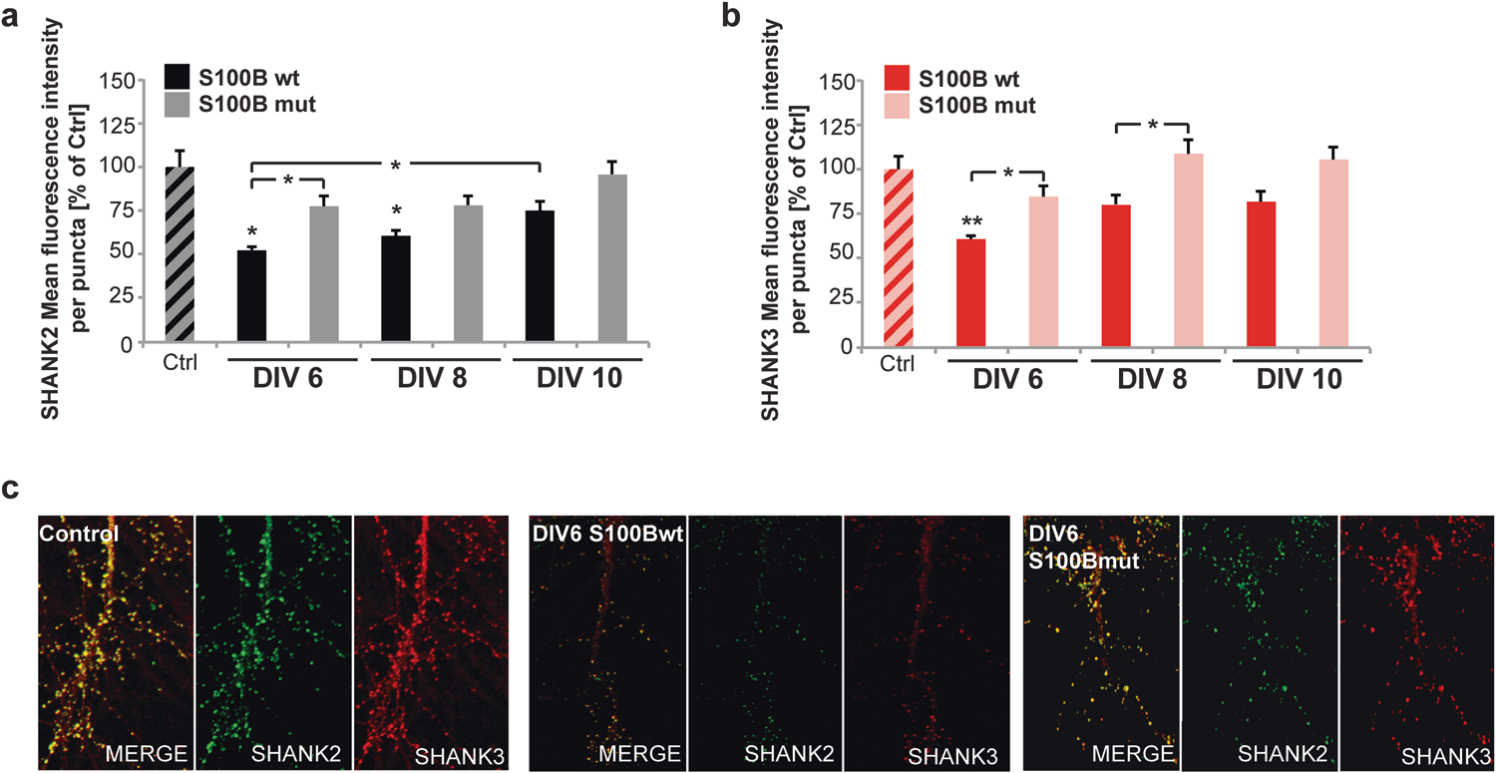
Chronic exposure to S100B has pronounced effects on developing synapses. **a, b** Primary hippocampal neurons were treated with either 30 μM S100Bwt or S100Bmut from DIV6, DIV8 or DIV10 until DIV11. **a** Treatment at early developmental stages (DIV6, DIV8) in combination with a prolonged exposure to S100Bwt but not S100Bmut led to a significant reduction in immunofluorescence intensities of SHANK2 immunoreactive puncta (*n* = 8–10 cells, Welch’s ANOVA, *F* = 12.518, *p* < 0.001; post-hoc analyses: control vs. wt_DIV6_, *p* < 0.05; control vs. wt_DIV8_, *p* < 0.05; control vs. mut_DIV6_, *p* > 0.05; wt_DIV6_ vs. mut_DIV6_, *p* < 0.05; wt_DIV6_ vs. wt_DIV10_, *p* < 0.05). **b** Decreased fluorescence intensity of SHANK3 immunoreactive puncta was seen in hippocampal neurons treated with S100Bwt protein from DIV6 or DIV8 on compared to untreated control (DIV6) or S100Bmut treated cells (DIV8) respectively. Non-zinc-binding S100Bmut protein did not affect SHANK3 fluorescence intensity independent of time-point of administration (*n* = 8–10 cells, one way ANOVA, *F*_(6,61)_= 7.7018, *p* < 0.001; post-hoc analyses: control vs. wt_DIV6_ *p* = 0.00111; wt_DIV6_ vs. mut_DIV6,_ *p* = 0.0952; wt_DIV8_ vs. mut_DIV8,_ *p* = 0.0227; control vs. mut_DIV6,_ *p* = 0.6094)_._ **c** Exemplary images of synapses of control neurons and neurons treated on DIV6 with S100Bwt or S100Bmut.

Previously, we have reported that the effects of zinc chelation regarding SHANK protein platforms at the PSD are more pronounced in young and immature synapses that initially contain the zinc-dependent SHANK2 and to lesser extent SHANK3, but not the zinc-independent SHANK1 [25]. In line with this observation, higher SHANK2 levels compared to SHANK3 were also found in this study in neuronal cultures treated at DIV10. Therefore, we investigated whether the application of S100B to an earlier developmental time-point in vitro produces more potent effects on SHANK protein levels at the synapse (Fig. 2). To that end, hippocampal neurons were treated starting at DIV 6, DIV 8, or DIV 10. DIV 6 marks the beginning of synapse formation in vitro, while at DIV 8, immature synapses are present. At DIV 10, the first mature synapses can be detected [41]. In contrast to previous results, our data showed that early exposure to S100B significantly affected both SHANK2 and SHANK3 levels (Fig. 2a, b). These effects were dependent on the zinc-binding of S100B, as S100Bmut did not provoke any changes in SHANK2/3 levels in these experiments. To rule out that the effects were caused by prolonged treatment, we also treated mature neurons at DIV 10 for 5 days (Supplementary Fig. S2f). S100B did not significantly decrease SHANK2/3 levels, and immediate effects observed after 24 h of treatment were compensated after five days since no chronic treatment was performed.

We conclude that persistently high levels of S100B can affect the stability of SHANK2/3 scaffolds at excitatory synapses in vitro via a zinc-dependent mechanism, which is pronounced in forming synapses. The sequestration of zinc into S100B leads to a reduction of synaptic SHANK2 and SHANK3. In line with this, we have previously observed a decrease of synaptic SHANK2 and SHANK3 by direct induction of zinc deficiency both in vitro and in vivo [25, 26].

### Increased levels of S100B in vivo during brain development lead to altered metal homeostasis in pups

In vivo, loss of synaptic *Shank* family members during embryonic development through zinc deficiency was accompanied by ASD-like behavior later in life [26, 35, 42], and *Shank2* and *Shank3* knockout (KO) mice are well-studied ASD mouse models [43, 44]. Therefore, given the effects of S100B on forming synapses, to investigate whether the observed alterations in zinc homeostasis and SHANK scaffold formation in vitro also occur during brain development in vivo, purified S100B protein [17] was intraperitoneally (ip) injected into pregnant mice during the last window of in utero development. Specifically, MYC-DDK tagged S100B or saline (from now on referred to as S100B and Ctrls, respectively) was administered to pregnant mice from embryonic day (E) 15 to E17 at the final experimental dose of 80 mg/kg.

**Fig. 3:**
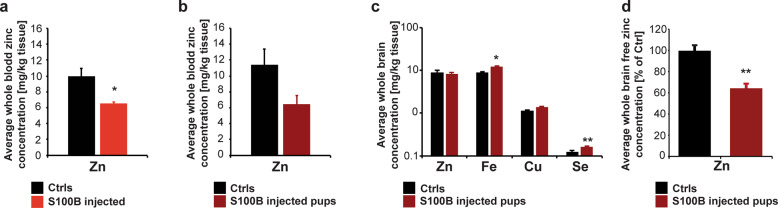
S100B injection alters zinc levels in vivo. **a**–**c** Measurement of trace element levels in whole blood and brain tissue of mice using ICP-MS. **a** The average whole blood concentration of Zn is significantly different between saline- and S100B-injected pregnant mice (*t*-test, *p* = 0.0335; *n* = 3 per group). **b** The average whole blood concentration of Zn is not significantly different between pups from Ctrls (*n* = 7), and S100B-injected mice (*n* = 4). **c** The average concentration of Zn and Cu in whole-brain tissue is not significantly different between pups from Ctrls (*n* = 10) and S100B-injected mice (*n* = 7). Fe and Se levels are significantly increased in pups from S100B-injected mice (*t*-test, *p*_Fe_ = 0.0356; *p*_Se_ = 0.0084). **d** The average free Zn concentration measured by Zinpyr1 fluorescence assays shows significantly lower free Zn levels in the brain of pups from mothers with S100B injection compared to Ctrls (*n* = 3 per group) (*t*-test, *p* = 0.0032).

In the first set of experiments, we analyzed whether S100B treatment alters trace metal homeostasis in the mothers and their pups (Fig. 3a, b). Our results showed that S100B-injected mice have significantly lower zinc levels in the blood (Fig. 3a). The alterations were specific for zinc, as no significant difference was detected for iron, copper, and selenium levels (Supplementary Fig. S3a). Thus, elevated S100B levels during pregnancy, either directly or by induction of secondary mechanisms, lower zinc levels in the blood of pregnant mice. As the developing embryo receives zinc through the placenta in exchange with maternal blood, we investigated whether lower zinc levels can also be detected in the blood of pups, averaging trace element levels from pups from different mothers. We found no statistically significant reduction in whole blood-zinc levels in pups from mothers receiving S100B injections compared to saline-injected controls (Fig. 3b). Also the levels of iron, copper, and selenium were not significantly different in the blood of pups from S100B-injected mice (Supplementary Fig. S3b).

Given that serum zinc levels are part of the rapidly exchangeable zinc pool and may change quickly, we investigated whether the reduction of maternal blood-zinc leads to lower zinc levels in the developing brain, a tissue with a slower turnover of zinc. While metal levels did not change significantly in the brain of the S100B-injected mothers (Supplementary Fig. S3c), pups from S100B-injected mothers showed altered trace metal profiles in the brain with a significant increase in total iron and selenium (Fig. 3c). While the total zinc levels measured using ICP-MS were not altered, the levels of free and weakly bound zinc measured by Zinpyr1 fluorescence were significantly decreased in the brain lysate from pups from S100B-injected mothers (Fig. 3d).

### Increased levels of S100B in vivo during brain development lead to a decrease in zinc-dependent Shank proteins at the synapse of pups

**Fig. 4:**
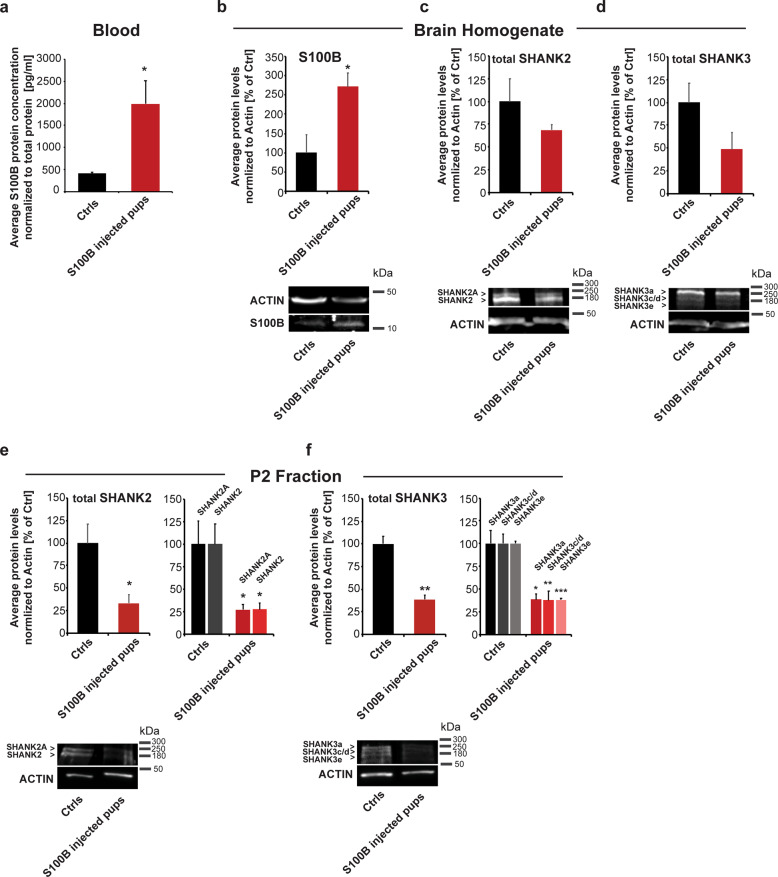
Elevated S100B levels in blood and brain of mice exposed to S100B in utero and decreased synaptic SHANK2 protein concentrations. **a** Increased blood S100B levels were detected in pups using S100B specific Luminex MAGPIX assays and normalized to the sample’s total protein concentration measured by BCA assays (*t*-test, *p* = 0.0343). **b** Western blot analysis of whole-brain lysate of pups from Ctrls and S100B mothers reveals significantly higher levels of S100B in the brain of pups exposed to S100B (*t*-test, *p* = 0.0432; *n* = 3 per group). **c, d** Western blot analysis of whole-brain homogenate of pups from Ctrls and S100B-injected mothers reveals no significant changes in SHANK2 (**c**) and SHANK3 (**d**) in the brain of pups exposed to S100B. **e, f** Using crude membrane (P2) fractions, we detected significantly lower synaptic SHANK2 levels (**e**) in S100B pups compared to Ctrls (*t*-test, *p*_SHANK2_ = 0.046; *n* = 3 per group). Both isoforms (SHANK2A and SHANK2) were similarly affected (right panel (*t*-test, *p*_SHANK2A_ = 0.0487; *p*_SHANK2_ = 0.0362; *n* = 3 per group)). **f** The levels of total SHANK3 were significantly reduced (*t*-test, *p*_SHANK3_ = 0.0018; *n* = 4 Ctrls, *n* = 3 S100B) in S100B pups compared to Ctrls. All higher MW isoforms (SHANK3a, SHANK3c/d and SHANK3e) were similarly affected (right panel (*t*-test, *p*_SHANK3a_ = 0.0487; *p*_SHANK3c.d_ = 0.0362; *p*_SHANK3c.d_ = 0.0362)).

Interestingly, pups from S100B-injected mothers had increased S100B levels in the blood on day one after birth (Fig. 4a), determined by Luminex Discovery and BCA assays. In pups’ brains, significantly higher protein content of S100B was detected in pups of mothers injected with S100B (Fig. 4b).

Given that significant alterations in trace metal homeostasis were detected in pups’ brains, we next investigated whether SHANK protein levels are altered in the brain of pups from mothers injected with S100B 24 h after birth. We could not detect a significant difference in SHANK2 or SHANK3 levels in whole-brain homogenate (Fig. 4c, d). However, it has been reported before that SHANK proteins undergo a shift from a synaptic PSD bound pool to a soluble pool under zinc-deficient conditions, affecting SHANK localization more than the total concentrations [25, 26]. Therefore, we analyzed crude membrane (P2) protein fractions (Fig. 4e, f). A significantly lower level of SHANK2 and SHANK3 was detected in whole-brain P2 fractions of mice from mothers exposed to high S100B levels during pregnancy. All higher MW isoforms detected (SHANK2A, SHANK2, and SHANK3a, SHANK3c/d, SHANK3e) were similarly affected in pups from mothers injected with S100B (Fig. 4e, f).

### Pups from mothers exposed to high S100B levels during pregnancy show behavioral alterations

Next, we wanted to evaluate whether the observed molecular phenotype of pups from mothers exposed to elevated S100B levels during pregnancy has a functional relevance on the behavioral level. Ctrls and S100B-injected mice gave, on average, birth to the same number of pups (7.33 ± 0.33 SEM and 7.67 ± 0.92 SEM, respectively) without any significant difference in pup weight between the groups at postnatal (P) day 21 (Ctrls: 8.21 ± 0.82 g SEM; S100B: 8.65 ± 0.93 g SEM). Furthermore, the injection did not result in significant body temperature changes hinting at no generalized immune response in the mothers in response to saline or S100B (Ctrls_before inject._ 35.9 ± 0.5 °C, Ctrls_after inject._ 34.3 ± 0.6 °C; S100B_before inject._ 35.8 ± 0.5 °C, S100B_after inject_. 35.8 ± 0.2 °C average body temperature measured four times per day, before injection and 2, 5, and 8 h after injection at E15; similarly, no significant changes in temperature were measured at E16 and E17).

**Fig. 5:**
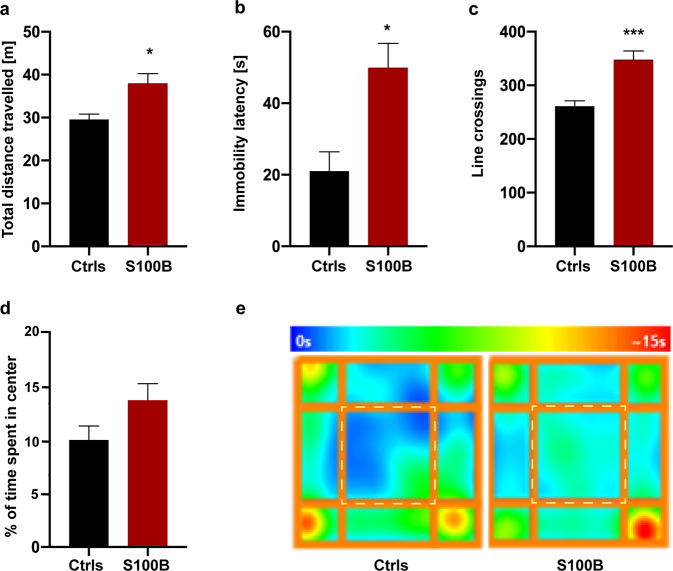
S100B injection during late embryogenesis induces motor hyperactivity in the adult. The motor performance and anxiety-like behavior were assessed in the open field task (Ctrls, *n* = 15; S100B, *n* = 36): **a** S100B mice show increased total distance (*p* = 0.045), (**b**) increased latency to the first immobility episode (*p* = 0.012) and (**c**) higher number of line crossings (*p* < 0.001) compared to Ctrls. **d** No significant difference was detected for the time spent in the center zone between the two groups (*p* = 0.126). **e** Occupancy plots of the two groups of mice. The center zone is indicated by the dashed square. Statistical analysis according to Mann–Whitney U test.

Given that ASD-associated behaviors and co-morbidities such as hyperactivity, anxiety, and cognitive impairment have been observed in *Shank2* KO*, Shank3* KO, and prenatal zinc-deficient mice [35, 43], in the first set of experiments, we tested the locomotor performance and anxiety-related behaviors in the open field (OF) test [45] (Fig. 5). Our results demonstrate that pups from mothers exposed to high S100B levels during pregnancy are hyperactive in the OF. Compared to Ctrls, mice from S100B-injected mothers showed a significantly higher locomotor activity and immobility latency (Fig. 5a, b) as well as an increased exploratory like-behavior as demonstrated by the total number of line crossings (Fig. 5c). Moreover, S100B mice covered the entire area of the OF without any discrimination between darker (more protective) or more lighted (more exposed) zones (periphery and center, respectively) as displayed in the occupancy plot (Fig. 5e). However, S100B mice did not show enhanced anxiety-like behavior, evidenced by the absence of significant difference in % of time spent in the central zone (Fig. 5d) of the OF and the lighted zone of the shuttle box in the light–dark test (Supplementary Fig. S4a).

**Fig. 6:**
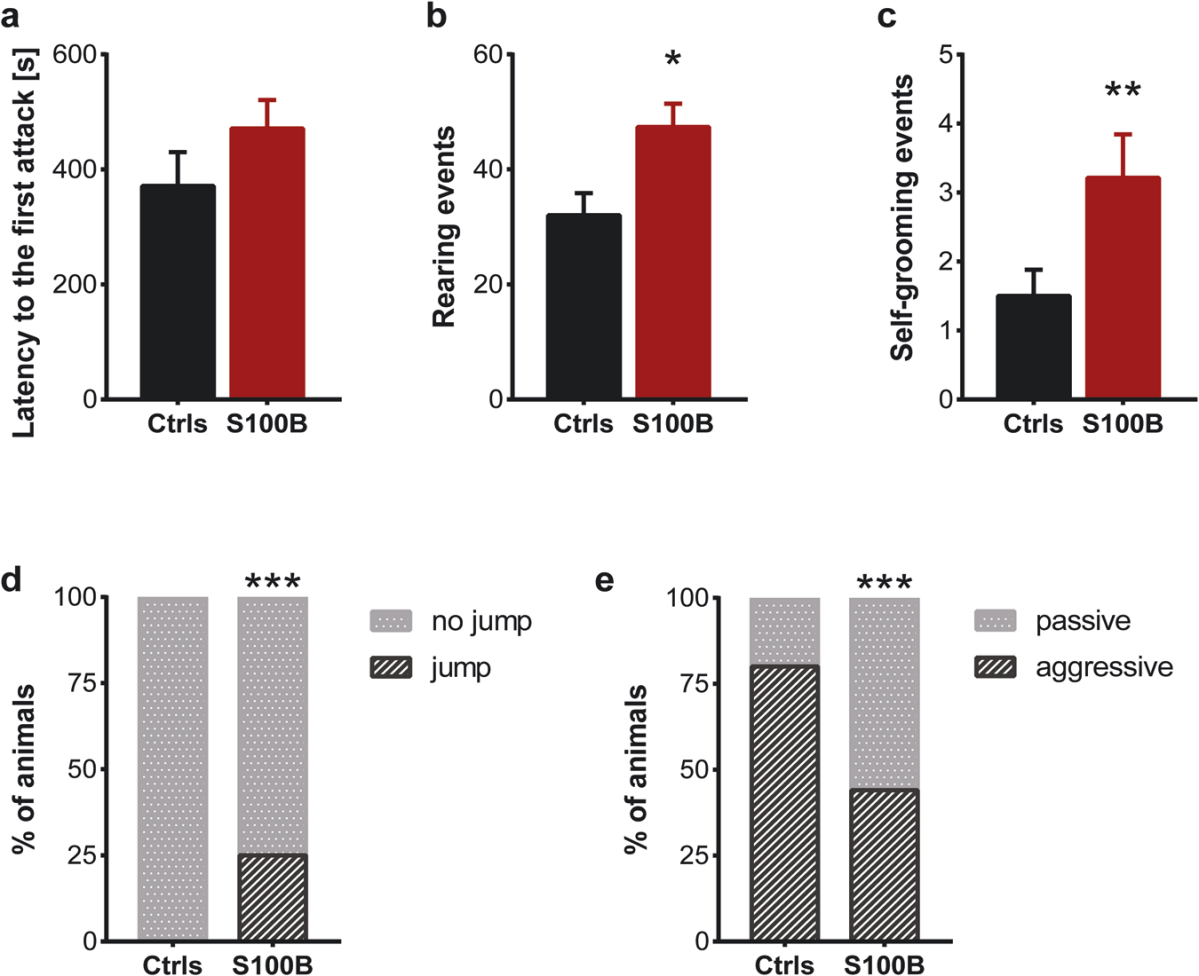
Adult mice that received S100B injection during late embryogenesis show social impairments and increased stereotypic behaviors. In the resident-intruder task, although (**a**) the latency to the first attack is not different between the two groups (*p* = 0.202), the percentage of mice that does not display an aggressive behavior during the interaction with the intruder is statistically higher in S100B group (**e**, Fisher’s test, *p* < 0.001). A significant increase in stereotypic behaviors such as (**b**) rearing (*p* = 0.021) and (**c**) self-grooming (*p* = 0.008) events were detected in S100B mice during both intruder (not shown) and marble burying tests. In addition, (**d**) the percentage of mice with jumping behavior is statistically higher in the S100B group (**f**, Fisher’s test, *p* < 0.001). Statistical analysis according to Mann–Whitney U test (when not indicated) or Fisher’s test.

To assess whether S100B treatment during pregnancy causes any alteration in social interaction and aggressive behaviors, mice were tested in a resident-intruder task. The latency to the first attack (Fig. 6a) and the total number of attacks or sniffing events (not shown) against the intruder were not statistically different between the two groups. However, an increased proportion of mice without aggressive behavior during the entire observational time was noticed in the S100B group (Fig. 6e).

Enhanced repetitive behavior is another core feature of ASD. Therefore, we analyzed stereotyped behaviors in two different stressful contexts, namely the exposure to a novel environment (first 10 min of marble-burying test habituation phase) and during social interaction (resident-intruder test, also during the habituation phase) [46]. Our results showed a significantly increased number of rearing, self-grooming, and jumping behaviors (though not of digging, not shown) in S100B mice during the habituation phase of both marble-burying and resident-intruder tasks (Fig. 6b, c, d), indicative of an increase in stereotypic behaviors. However, no significant differences were detected between the two groups in the latency to bury the first marble (Supplementary Fig. S4b) and the total number of buried marbles (Supplementary Fig. S4c), suggesting no changes in compulsive-like behavior.

**Fig. 7:**
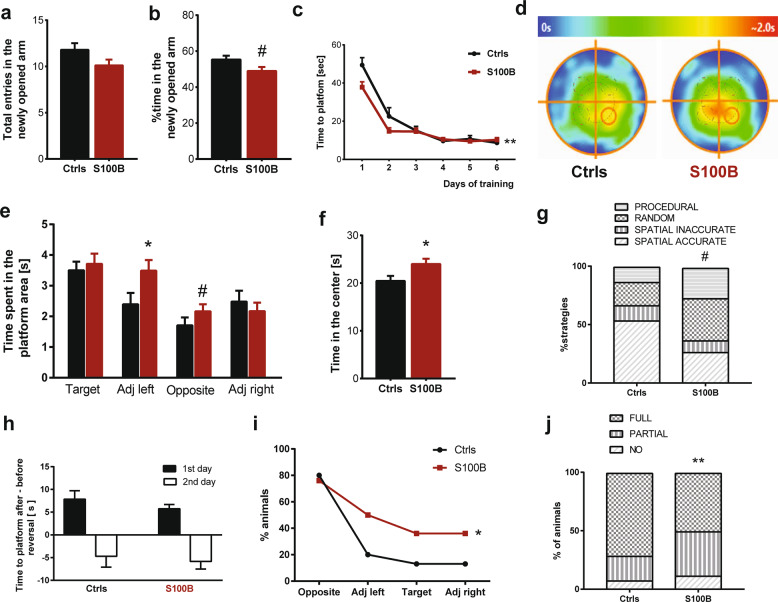
Adult mice that received S100B injection during late embryogenesis show cognitive deficits. **a** No significant difference was observed in the entries into the newly opened arm between S100B and Ctrl mice (*p* = 0.148) while (**b**) a trend for a significant decrease in time spent in the newly opened arm was observed in S100B mice (*p* = 0.092). **c** Analysis of the learning curve showed a significant effect of S100B exposure (repeated-measures ANOVA, Time *F* (5, 43) = 80.862, *p* < 0.001, Treatment *F* (1, 43) = 3.715, *p* = 0.061, Time × Treatment *F* (5, 43) = 3,340, *p* = 0.006). **d** Heat map of the time passed by the Ctrls and S100B mice in the maze during the probe test. **e** S100B mice spent more time in the adjacent left (*p* = 0.042) and opposite (*p* = 0.065) sectors. **f** S100B mice spent more time in the maze center (*p* = 0.033). **g** The analysis of search strategy showed an increase in non-spatially targeted strategies in S100B mice (Chi-square test, *p* = 0.057). **h** No significant difference between S100B and Ctrls was shown for the time to reach the platform in the reversal test, but (**i**) a significantly higher percentage of S100B mice continued to target the old platform location before finding the platform in the new location on the second day of the test (log-rank test, *p* = 0.037; note that the quadrants indicated are referred to the new location of the platform). **j** The percentage of S100B mice building only a partial nest structure is significantly higher than that of Ctrl mice (*p* = 0.009, Fisher’s test; Ctrls, *n* = 15; S100B, *n* = 36). Statistical analysis according to Mann–Whitney U test (when not indicated), Fisher’s test, Chi-square test. Log-rank test or repeated-measures ANOVA.

Lower intellectual and cognitive abilities are a frequent observation in individuals with low-functioning autism, and impaired learning and memory abilities have been previously observed in ASD mouse models [47]. Therefore, next, to assess if S100B mice show impaired learning and memory, we evaluated their performance in the Y-maze novel arm and Morris water maze (MWM). In the Y-maze novel arm test, during the second session when all arms are open, we did not observe a significant difference either in the total number of entries into the three arms (not shown) or in the entries into the newly opened arm (Fig. 7a) between S100B and Ctrls mice. However, the time spent in the newly opened arm shows a trend toward a significant reduction in S100B mice (Fig. 7b). The results did not evidence major spatial or working memory deficits of S100B mice in this test, while the trend toward a significant reduction in the time spent in the newly opened arm of the Y-maze test may suggest restricted behavioral flexibility/patterns.

The mice were also tested for their ability to find a hidden platform in the MWM for 6 days and were subjected to the probe test 24 h after the last training session (day 7) and to the reversal test on days 8 and 9. The learning curve analysis showed that S100B mice have an initial acceleration of the learning phase but reached an identical level of performance after repeated training (Fig. 7c). The probe test evidenced a different search strategy of S100B with respect to Ctrls. As shown by the heat map (Fig. 7d), S100B mice have a non-targeted search strategy that brings them to sample the center of the maze with a modest preference for the sector where the platform had been previously located. In fact, S100B mice spent significantly more time searching for the platform in the sector that is adjacent to the correct sector (Fig. 7e), and a trend for a significant increase is also present for the opposite sector, and searching for a significantly longer time in the center zone of the maze (Fig. 7f). Accordingly, the analysis of searching strategies hints at random and procedural strategies of S100B mice compared to the spatially-oriented strategies of Ctrls (Fig. 7g). No difference was observed in the time to reach the new platform location at the reversal test confirming a preserved learning ability in S100B mice (Fig. 7h). However, a significantly higher fraction of S100B mice persisted in reaching the old platform location before finding the platform in the new location on the second day of the reversal test (Fig. 7i). While the initial improvement in learning performance tallies well with the increased exploratory activity observed at the OF test, the impairment in the probe test points to moderate impairment in spatial memory (probe test) and perseveration behavior (reversal test) in S100B mice.

Finally, we evaluated general cognitive abilities in S100B mice, assessing their capacity to build nests. The nest-building test revealed a significant increase in the % of mice building only a partial nest in S100B mice compared to Ctrls (Fig. 7j).

## Discussion

Apart from genetic factors, immune- and neuroinflammatory processes may play an essential role in the pathology of ASD. Several studies could show an increased expression of pro-inflammatory cytokines in the brain and CSF of patients with ASD [48–52]. Multiple studies could also identify maternal infection during pregnancy as a risk factor for the development of ASD [53–57]. Maternal immune activation (MIA) in mice, often produced by injection of lipopolysaccharide (LPS), acts as a nongenetic factor in the etiology of ASD via elevated IL-6 levels [58].

Interestingly, S100B secretion can be enhanced by the pro-inflammatory cytokine IL-6 [14]. Besides the ability to bind calcium, S100B can bind two zinc ions per homodimer [17, 59, 60]. Intriguingly, prenatal zinc supplementation reduced the stress response observed in adult offspring from rats exposed to LPS during gestation [61] and prevented communication impairments and social autistic-like behaviors [62, 63] in the offspring of MIA mice, as well as reduce pro-inflammatory signaling [64], suggesting a clear link between inflammation and zinc homeostasis. Thus, we investigated whether crosstalk between high S100B levels and zinc deficiency exists, acting on known ASD-associated pathways at excitatory synapses, thereby linking hypozincemia, inflammation, and known genetic factors in a single synaptic pathomechanism of ASD.

In this study, we modeled increased levels of S100B during the last trimester of pregnancy, a critical time window in brain development of the embryo. High levels of S100B have been reported in ASD and may also occur in response to immune system activation. While S100B has been reported to possess important protective functions, chronically elevated levels may result in additional pathophysiological effects.

The increase in S100B levels resulted in a significant reduction of maternal blood-zinc levels. We could further observe that altered maternal zinc levels lead to decreased free zinc levels in pups’ brains that we measured directly after birth. In addition, the metallome in the brain of developing embryos was disturbed with an increase of total iron and selenium in brain tissue. Given that trace elements influence each other leading to the establishment of trace metal profiles, it is likely that reduced zinc levels will also affect the balance of other metals [65].

Our study shows that overload with S100B during the time window of synapse formation also reduces zinc-dependent proteins of the SHANK family in vitro and in vivo. This was previously similarly shown by the induction of copper overload during brain development, causing a secondary zinc deficiency [40] and by zinc deficiency directly [26]. All synaptic higher MW isoforms of SHANK2 and SHANK3 were affected in this study. According to the literature, all these isoforms contain the zinc-binding C-terminal SAM domain [28, 66].

The loss of the zinc-sensitive SHANK proteins SHANK2 and SHANK3, notwithstanding whether caused directly by zinc deficiency or indirectly, may affect synapse function with possibly lasting effects on brain function later in life [24]. Besides the many functions of zinc, its modulatory effect on SHANK proteins may be a major contributor to the etiology of ASD as mutations in *Shank* family members have been frequently identified to result in ASD in humans [29] and mouse models [44]. In vitro, the loss of synaptic SHANK proteins was a direct consequence of zinc scavenging by S100B. The number of synaptic signals was not affected. However, chronically low SHANK2/3 levels due to prolonged exposure to S100B likely will affect synapse density as reported previously [25].

In vivo, we have detected elevated levels of S100B in the blood and brain tissue of pups from S100B-injected mothers. Given that brain S100B was not detected by anti-DDK tag antibodies, maternal S100B exposure may have induced a prolonged increased expression of endogenous S100B in pups. It has been shown for some tissues that S100B/RAGE interaction activates NF-κB and induces pro-inflammatory mediators, among them IL-1, IL-6, TNFα, and S100B [67], thus creating a feedback that may be the basis for high S100B levels. Systemically, S100B is excreted and eliminated exclusively through the kidneys [68]. However, it is unknown whether the S100B bound zinc is recycled. Future studies will need to explore whether injected maternal S100B is transported into the fetal system, but its low MW makes it possible that S100B crosses the placenta [69]. Besides, within the brain, S100B has an autoregulatory function. S100B can stimulate its release via binding to RAGE receptors [70]. Thus, small initial increases of S100B levels may drive a further and more lasting elevation of S100B.

Previous studies using transgenic mice overexpressing S100B reported impairments in hippocampal LTP and spatial learning [71]. Although in this study we did not measure LTP, SHANK protein levels have been shown to have a regulatory function in LTP [72], and the effects of S100B on LTP may, in part, be mediated by their influence on SHANK3 proteins via modulation of zinc homeostasis. However, the mice with transgenic overexpression of S100B have a chronic elevation of S100B and not only experienced elevated S100B levels in utero. In contrast, S100B KO mice have enhanced hippocampal synaptic plasticity and HIP-dependent learning and memory [73].

Along with the molecular changes, offspring of mice exposed to high S100B levels during pregnancy also show behavioral abnormalities. As also reported for *Shank2* KO [43, 74], mice that received S100B during synapse development show hyperactivity as a prominent feature; in addition, similarly to *Shank2* KO mice, S100B mice also displayed increased repetitive behaviors, such as jumping, rearing, and self-grooming [43, 74], impaired nest-building but normal working memory [43], as evaluated at the Y maze test. Increased self-grooming was also reported in several *Shank3* KO mice [43, 75–78]. However, hyperactivity and increased repetitive behaviors were not reported in KO mice for the non-zinc binding SHANK family member *Shank1* [79–81]. Regarding spatial memory, impaired learning abilities in the MWM were previously reported for one isoform-specific *Shank3* KO mouse model [77], though not for other *Shank3* KO mice [75, 76, 78]. Impairment in cognitive abilities in S100B mice was also demonstrated by their decreased ability of nest-building. Overall, these behavioral impairments can be interpreted as a feature of ASD and have been observed in other mouse models with ASD-like phenotype [82, 83]. In addition, as in our S100B model, disabilities in social behavior were noted in *Shank2* and *Shank3* KO mice and prenatal zinc-deficient mice [35]. However, unlike *Shank* KO mice or prenatal zinc-deficient mice, S100B exposed mice do not show increased anxiety behavior.

Nevertheless, these results hint toward largely shared behavioral phenotypes between the *Shank2* and *Shank3* KO mouse models for ASD and mice from mothers with high S100B levels during pregnancy that may be explained by the loss of SHANK2 in pups from these mothers. The absence of increased anxiety also hints at the presence of further modifying factors in S100B exposed mice or *Shank2* and *Shank3* KO mice that modulate the phenotype, such as extracerebral factors like gastrointestinal abnormalities that, for example, have been reported in *Shank3* KO mouse models [84–86].

In conclusion, the data presented in this study reveals how CNVs affecting *S100B* and high S100B levels that have been reported in ASD may mechanistically be linked to known pathophysiological processes of ASD. S100B proteins that increase in pro-inflammatory situations create a link between immune system activation, trace metal dyshomeostasis, and synaptopathies in ASD. Mediating the crosstalk between inflammatory processes, hypozincemia, and the effects on the NRXN-NLGN-SHANK pathway is at the center of ASD synaptic impairments [34], S100B proteins may be important drug targets for treatment strategies in ASD.

## Material and methods

### Reagents

Zinpyr-1 was purchased from Sigma-Aldrich. Primary antibodies were obtained from Sigma-Aldrich (Map2, S100B), Synaptic Systems (Shank3, S100B), Biomol (S100B), and OriGene (DDK). Shank antibodies have been described previously [26, 43]. Secondary antibodies (Alexa488, Alexa568) were purchased from Life Technologies. Unless indicated otherwise, all other materials, chemicals and reagents were purchased from Sigma-Aldrich.

### Hippocampal culture from rat brain

The preparation of hippocampal cultures from E18 rat brains was performed as described previously [41]. After tissue extraction and processing, the hippocampal neurons were seeded on poly-l-lysine (PLL) (0.1 mg/ml; Sigma-Aldrich) coated glass coverslips in a 24 well plate at a density of 3 × 10^4^ cells/well. Alternatively, cells were seeded in a 10 cm Petri dish at a density of 2.5–3 × 10^6^ cells/dish. Cells were maintained in Neurobasal^TM^ medium (Life Technologies), with added B27 supplement, 0.5 mM L-Glutamine, and 100 U/ml penicillin/streptomycin (all from Life Technologies) and grown at 37 °C in 5% CO_2_.

### Treatment of hippocampal cells

For the analysis of synaptic proteins, neurons were treated with 30 μM S100B or 30 μM S100Bmut protein at DIV10 for 24 h or with 30 µM S100B 1 h pre-incubated with 60 µM ZnCl_2_ on ice at DIV 8 for 24 h.

### Mutagenesis of zinc-binding amino acids of S100B

The non-zinc-binding mutant of S100B was published previously [17]. In brief, pLenti S100b vector (OriGene) was used for the mutagenesis of S100B zinc-binding sites and carried a C-terminal Myc-DKK tag. This Myc-DKK tagged construct is referred to as S100Bmut here. The tagged wild-type (WT) and zinc mutant were subsequently cloned into a pGEMEX expression vector for protein production in *E. coli*.

### Recombinant expression and purification of S100B

Human S100B WT was expressed in *E. coli* and purified using previously established protocols [87] either in non-tagged or Myc-DKK tagged versions [17]. Briefly, the Zn S100B mutant was purified with a strong anion exchange chromatography (HiPrep Q FF 16/10, GE Healthcare), followed by gel filtration (HiLoad 16/600 Superdex 75, GE Healthcare) and finalized with another strong anion exchange chromatography (HiPrep Q FF 16/10, GE Healthcare). Demetallated forms of S100B and S100Bmut were generated by incubating the protein with 0.5 mM EDTA and 300-fold excess of DTT for 1 h at 37 °C, followed by elution from a gel filtration column (24 mL S75 Tricorn, GE Healthcare).

### Immunodetection

*Immunocytochemistry:* For immunofluorescence, primary cultures were fixed with 4% paraformaldehyde (PFA)/4% sucrose/PBS at 4 °C for 20 min and processed as follows. After washing 2 × 5 min with 0.2% Triton-PBS at room temperature (RT), blocking was performed with 10% fetal bovine serum (FBS) in 1× PBS for 1 h at RT, followed by the primary antibody at RT for 2 h. After a 3 × 5 min washing-step with PBS, cells were incubated with the secondary antibody AlexaFluor 488 or 568-conjugated for 1 h at RT. Cells were then rewashed in PBS for 10 min, counterstained with DAPI for 5 min, and mounted with Vecta Mount (Vector labs).

*Fluorescent measurement -* All images were evaluated under “blinded” conditions. Fluorescence images were taken with an upright Axioscope microscope equipped with a Zeiss CCD camera (16 bits; 1280 × 1024 ppi) and Axiovision software (Zeiss), and evaluated with ImageJ 1.51j.

### Cell health assay

Primary hippocampal neurons were seeded at a density of 5000 cells per well on PLL coated E-Plate VIEW 16 plate (ACEA Biosciences, San Diego, USA). At DIV10, cells were treated with 0.1 µM, 1 µM, or 30 µM S100B protein or 5% DMSO as a positive control for 24 h. Impedance was measured every 15 min within the first 6 h of treatment and every 30 min in the following 18 h of treatment using the xCELLigence RTCA Systems (ACEA Biosciences, San Diego, USA). A decrease in impedance is associated with the detachment of cells and, therefore, a sign of neuronal cell death.

### Animals

For primary hippocampal cell cultures, pregnant rats were purchased from Janvier Labs and were housed in plastic cages under the standard laboratory conditions (average temperature of 22 °C, food, and water available ad libitum). Lights were automatically turned on/off in a 12 h rhythm (lights on at 7 am).

For the generation of an in vivo model of S100B overexposure, C57Bl6/J pregnant mice were daily ip injected with 80 mg/kg S100B or saline from E15 to E17 (the presence of the plug was considered as E0.5). The first cohort of mothers and pups, after birth, was sacrificed, and blood and brain were collected for further analysis. The second cohort of pups was used for behavioral and histological analysis. Samples sizes were chosen according to published experimental procedures for tests performed. Animals were randomly assigned to treatment and control groups.

Mice were kept in a conventional animal facility with controlled temperature (20–24 °C) and humidity (60%) on a light/dark cycle of 12 h. A total of six pregnant mice were housed in standard cages (2 mice/cage) with nesting materials; at P24, sex was identified, and pups were housed in standard cages (six mice/cage) in the absence of physical and structural environmental enrichments [88]. Food and water were available ad libitum, and body weight was recorded throughout the entire observation period. All animal procedures were approved by the Committee on Animal Health and Care of the University of Modena and Reggio Emilia (authorization number: 913/2018PR), the Ulm University (ID Number: O.103), and conducted following National Institutes of Health guidelines.

### Measurement of trace metal concentrations

*In cells:* For fluorescent Zn-staining of cultured neurons, growth medium was discarded, and the cells were incubated with a solution of 5 μM Zinpyr1 in PBS for 1 h at RT. Afterwards, coverslips were washed with DAPI-PBS and mounted with Vecta Mount.

*In tissues:* Sample preparation: Blood samples (20 μl) from mice and the control material Seronorm Trace elements Whole blood L-1^®^ (20 μl) were weighed into Eppendorf vials, 10 μl yttrium solution (1 mg Y/l) as internal standard was added and the solutions were digested with 50 μl nitric acid (70%, ACS, Sigma Aldrich, UK) on a shaking incubator at 95 °C for 30 min in closed vials. After 30 min, 50 μl hydrogen peroxide (30%, ACS, Sigma-Aldrich, UK) was added, and the mixture was again heated for 30 min at 95 °C. The samples were cooled and filled up to 1 g with 18 MΩ cm water.

Tissue homogenates from mouse brains were digested using Proteinase K for 1 h at 37 °C. The resulting solutions were weighed into Eppendorf vials, 10 μl yttrium solution (1 mg Y/l) as internal standard was added, and the solutions were further digested with 50 μl nitric acid (70%, ACS, Sigma Aldrich, UK) on a shaking incubator at 95 °C for 30 min in closed vials. After 30 min, 50 μl hydrogen peroxide (30%, ACS, Sigma-Aldrich, UK) was added, and the mixture was again heated for 30 min at 95 °C. The samples were cooled and filled up to 1 g with 18 MΩ cm water. Control material for the brain digests were Seronorm Trace elements Whole blood L-1^®^ and Seronorm Trace elements Serum L-1^®^. Inorganic Ventures 71 A standard (multielement 10 mg/l standard, Inorganic Ventures, USA) was diluted appropriately in 5% (v/v) nitric acid.

Instrument set-up and measurement: The samples were measured with an 8800 ICP-MS/MS from Agilent (UK) using hydrogen (4 ml H_2_/min) as a reaction gas in MS/MS mode to remove interferences. An AS110 with a micro nebulizer (50 μl/min) was used for sample introduction. The system was optimized for sensitivity and resolution before the measurement. All samples were measured with ten replicates using ^89^Y as an internal standard. The analyzed isotopes were ^56^Fe, ^57^Fe, ^63^Cu, ^65^Cu, ^66^Zn, ^67^Zn, ^68^Zn, ^78^Se, and ^80^Se.

Data analysis: Element concentrations were calculated using external calibration. Control material for the blood samples was prepared as six independent samples. The material for the control measurement of the brain lysates was each prepared in triplicate. Blood samples from mothers were also prepared in triplicate. For these samples, the average and standard deviation were calculated. Standard deviation (SD) for the brain samples and the pups’ blood samples is the SD of the measurement.

*Zinpyr1 assay –* A cell-permeable fluorogenic Zn^2+^ reporter (0.5 mM stock solution in DMSO) was used (Zinpyr1, Abcam). Crude homogenates from mouse pup brains were diluted in ddH_2_O, following the sample dilutions 1:10, 1:25, and 1:50. 100 μl of samples were incubated with 50 μM Zinpyr1 at RT for 30 min in the dark. The assay was performed using an assay control: 1:10, 1:25, and 1:50 dilutions of a 5 mM ZnCl_2_ solution. Zinpyr1 fluorescence was monitored in a 96-well clear bottom, black-sided plate (Greiner Bio-One, North Carolina, US) on UVITEC Alliance Q9 imager using an excitation wavelength of 515 nm and an emission wavelength of 515 nm.

### Protein biochemistry

*Protein Fractionation -* To obtain P2 fractions from hippocampal cultures, DIV10 cells exposed to 30 μM of S100B or S100Bmut for 24 h were harvested and homogenized in homogenization buffer (320 mM sucrose, 10 mM HEPES, pH 7.4) containing protease inhibitor (Roche). Homogenate was further centrifuged at 3200 *rpm* for 15 min resulting in supernatant S1 (soluble fraction) and pellet P1 (membrane-associated fraction). Subsequently, S1 was centrifuged for 20 min at 11,400 *rpm*, resulting in S2 (soluble fraction) and P2 (crude membrane fraction). The resulting pellet P2 was resuspended in a homogenization buffer to perform protein quantification (Bradford assay) and analyzed by WB.

To obtain P2 fractions from brain tissue, 1 g tissue was homogenized in 10 ml Buffer A. Crude homogenate was centrifuged as described above. The resulting pellet P2 was resuspended in Buffer A to perform protein quantification (Bradford assay) and analyzed by WB.

*Western Blotting -* Proteins were separated by SDS-PAGE and blotted onto nitrocellulose membranes. Immunoreactivity was visualized using HRP-conjugated secondary antibodies and the SuperSignal detection system (Pierce, Upland, USA). Images were taken with a UVITEC Alliance Q9 imager. *Western blot quantification -* Evaluation of bands from WB was performed using ImageJ v1.49o. Three independent experiments were performed. Protein bands of interest were selected, and the integrated density was measured. All WB bands were normalized to a housekeeping protein, and the ratios averaged and tested for significance.

*Protein quantification using MAGPIX*^*®*^ *and BCA -* 5 µl of blood lysate was used for protein quantification using the Pierce BCA assay as per the manufacturers’ instructions (Thermo Fisher). The remaining volume of whole blood lysate was added to a Luminex Multiplex kit (LXSAHM-03 customized for S100B detection (R&D systems, MN, US)). Multiplex detection was carried out as per the manufacturers’ instructions. Briefly, samples and standards (standard concentration range: 40.4–9800 pg/ml) were added to a 96 well plate with a microparticle cocktail and incubated for 2 h at RT. The plate was placed on a magnet and washed with a wash buffer before incubating for 1 h with a biotin-antibody cocktail. The washing step was repeated, and the samples were then incubated for 30 min with PE-Streptavidin. After a final washing step, the microparticles were resuspended in 100 µl of wash buffer. The plate was read on the MAGPIX System (Luminex, TX, US). The quantity of S100B detected in the samples was corrected based on the volume added to the plate and normalized against the total protein as determined using the BCA assay to calculate the level of S100B / mg of total protein.

### RNA extraction and qRT PCR

RNA was isolated using the Qiagen RNeasy kit according to the manufacturer’s protocol. First-strand synthesis and quantitative real-time PCR were performed using the QuantiFast^TM^ SYBR_Green RT-PCR kit (Qiagen) in a one-step, 20 µl volume, single-tube format according to the manufacturer’s protocol with gene-specific QuantiTect Primers (Qiagen). Amplification and fluorescent detection of the SYBR Green I reporter dye were performed using a Rotor-Gene Q real-time PCR machine (model 2-Plex HRM) with Rotor-Gene Q Software (version 2.0.2) (Qiagen). All qRT-PCR reactions were performed in technical triplicates. The mean Cycle threshold (C_t_)-values for each reaction were used for calculations.

*qRT PCR quantification –* Relative quantification is based on the *hmbs* gene as internal reference to determine the virtual mRNA level of target genes. C_t_ values were transformed into virtual mRNA levels as follows: virtual mRNA level = 10 * ((C_t (target)_ – C_t(standard)_)/slope of standard curve).

### Behavioral analyses

All behavioral tests were performed by an operator unaware of the treatment to avoid bias. Animal behavior was recorded and automatically analyzed with ANY-maze Video Tracking system (Stoelting), and stereotypies were manually counted. Both male and female pups (Ctrls: 6 males, 9 females; S100B: 16 males, 20 females) were used; the same cohort of mice underwent different tests at different ages (see Table 1).

**Table 1:** Age distribution of mice for behavioral tests.

Age (months)	Test
2	Open field
4	Marble burying
6	Light–dark box
7	Y maze novel arm
8	Morris water maze
9	Resident-intruder
	Nest building

*Open field test -* The motor activity and exploration in a new environment were assessed in the OF test. Mice were placed in in the center of an open wooden chamber (50 × 50 × 40 cm) with dark walls and allowed to explore freely for 10 min. The open space was virtually subdivided into 16 adjacent squares and the number of times the line of a square is crossed with all four legs was recorded to assess locomotor activity. Moreover, the space was virtually subdivided into 2) a peripheral zone (within 10 cm of the walls), a central zone (the rest of the arena), and corners for assessment of anxiety. Traveled distance, total zone entries (line crossings), immobility behavior, and time spent in each zone were automatically recorded. The time spent in the center of the arena was considered as an anxiety index. In addition, the area covered by the animal body during the test was evaluated and reported as a heat-map of the cage occupancy. The apparatus was thoroughly wiped with 70% ethanol after each test to avoid olfactory cues.

*Marble burying test -* The marble burying test was performed to assess anxiety-like/compulsive behavior [89]. Each mouse was placed in a cage (36 × 25 × 25 cm) and, after 30 min of acclimation, 20 black marbles of 1.5 cm of diameter were equally distributed on the top of bedding material (5 cm in depth). Mice were allowed to explore for 15 min freely. During acclimation, the number of self-grooming, rearing, and digging events were recorded, while during the test, the number of buried marbles, and the latency to the first burying event, were counted. To avoid olfactory cues, each animal received fresh bedding material, and each marble was cleaned with 70% ethanol.

*Light*–*dark test -* To further explore anxiety-like behavior, the light–dark paradigm was used [90]. A shuttle box composed of a bigger (40 × 40 × 50 cm) lightened chamber with white floor and walls, and a smaller (15 × 40 × 50 cm) shaded chamber with black floor and walls was employed. Animals were placed in the light chamber and allowed to explore the whole apparatus for 15 min. The latency to the first entry, the number of entries and the time spent in the dark zone, and the frequency of self-grooming and rearing, and the percentage of time spent in the light zone, were recorded. The apparatus was thoroughly wiped with 70% ethanol after each test to avoid olfactory cues.

*Y maze novel arm test -* The ability to discriminate similar contexts and explore a new environment was tested in a Y maze [91]. The maze was constructed of black plastic walls (22 × 7 × 20 cm), and three arms were connected through a symmetrical three-way central corridor. The test was performed on 2 consecutive days: on day 1, a 15 min acclimation was conducted by placing the mouse in the Y maze with one of the three arms closed (novel arm). The mouse was placed into the Y maze with the closed arm on the following day and allowed to explore for 15 min freely. Then, the novel arm was opened, and the animal could move freely for 5 min. For each trial, the time and the number of entries in each arm were recorded. The apparatus was thoroughly wiped with 70% ethanol after each test to avoid olfactory cues.

*Morris water maze test* - The MWM test was used to assess mouse spatial learning and memory [92]. Animals were placed in a circular pool (75 cm diameter) filled with water at 19 °C, made opaque by adding white non-toxic paint, and allowed to swim for 60 s or until they found the location of a hidden circular platform with 11 cm diameter. The pool was virtually divided into four quadrants, characterized by four visible spatial cues different in color and shape. During the learning phase, mice were trained with four trials per day (starting from a different quadrant at each trial) for 6 days with 60 min inter-trial interval. For each trial, the time to reach the platform, the total distance traveled, and the mean speed was evaluated. As a learning index, we calculated (1-(time to target at 6th day/time to target at the first day)). On the 7th day, the platform was removed, and the animals were allowed to swim for 60 s (probe test), and the % of time spent in the quadrant with the removed platform was evaluated. On the 8th and 9th day, the platform was placed in a new position (reversal phase), and mice were tested with four trials/day similarly to the above-described procedure; the time spent in the old-platform quadrant along with the time to reach the new platform was recorded. The analysis of target search strategy was carried out according to current literature [93]. Briefly, spatial accurate strategy and non-spatial strategy were distinguished considering individual swim paths during the probe test.

*Resident-intruder test -* To evaluate aggressive behavior, male mice were subjected to the resident-intruder paradigm [94]. Experimental animals (residents) were housed in transparent cages (36 × 25 × 25 cm) singly for 24 h before the test to develop territoriality. The test was performed by introducing an unfamiliar male mouse (intruder) into the resident home cage. Mouse performance was recorded for 10 min, and the number of attacks and the latency to the first attack were counted only for aggressive residents while sniffing, and stereotypies were also counted for passive residents.

*Nest building -* To evaluate general wellness [95] and hippocampal function, mice were housed singly in small (20 × 15 × 15 cm) transparent cages and provided with 6 g of compacted cotton to allow nest building. The formation of the nest was evaluated 18 h after cotton deposition and scored as full, partial, or no nest according to the cotton usage.

*Statistics-* Statistical analysis was performed using one-way analysis of variance (ANOVA) with an appropriate post-hoc test, such as Tukey test or nonparametric Mann–Whitney U test, Fisher’s test, or repeated-measures ANOVA, using the statistical package SPSS (version 26). Significance thresholds were set at *p* < 0.05, with *p* < 0.05*; <0.01**; <0.001***.

## Supplementary information

Supplementary Figure legends

Figure S1

Figure S2

Figure S3

Figure S4
