# Supplementary material for: S100B dysregulation during brain development affects synaptic SHANK protein networks via alteration of zinc homeostasis
Source: Transl Psychiatry. 2021 Nov 5;11:562. doi: 10.1038/s41398-021-01694-z (PMC8571423; doi:10.1038/s41398-021-01694-z)
Supplement: Supplementary file 1 — Supplementary Figure legends [file 41398_2021_1694_MOESM1_ESM.docx]

**S100B dysregulation during brain development affects synaptic SHANK protein networks via alteration of zinc homeostasis**

Eleonora Daini, Simone Hagmeyer, Chiara A. De Benedictis, Joana S. Cristóvão, Martina Bodria, Aisling M. Ross, Andrea Raab, Tobias M. Boeckers, Jorg Feldmann, Cláudio M. Gomes, Michele Zoli, Antonietta Vilella, Andreas M. Grabrucker

**Suppl. Figure Legends:**

**Supplementary Fig. S1: S100B is expressed predominately in the cerebellum and enriched towards synaptic sites. a,b)** mRNA and protein lysate was prepared from mouse brain (n = 3). **a)** Endogenous S100B expression can be detected in all four brain regions: cortex (CTX), hippocampus (HIP), striatum (STR), and cerebellum (CER). The expression of S100B mRNA is highest in the CTX and CER, with high inter-individual variability in CER. Among CTX, HIP and STR, expression levels are significantly higher in CTX compared to the other two brain regions (one-way ANOVA, F_2,6_ = 39.725, *p* < 0.0001, Post-hoc analysis: CTX vs HIP: *p* = 0.001; CTX vs. STR: *p* = 0.0016). **b)** On the protein level, fractionation experiments using brain homogenate (Ho), soluble protein fractions (S2), and crude membrane fractions (P2) show equal distribution of the S100B protein. As control, SHANK2, as synaptic membrane associated protein, shows significant enrichment in P2 fractions confirming successful fractionation. **c)** Hippocampal neuron labeled with an anti-DDK antibody detecting exogenously applied S100B only and anti-SHANK2 antibody as a synaptic marker. Several S100B immunoreactive puncta co-localize with the synaptic marker. Merged images show additional DAPI staining of the nucleus.

**Supplementary Fig. S2:** **a-e)** Treatment of hippocampal cultures with 30 μM S100B for 24 h. **a)** No significant decrease in the cell index measured by xCELLigence RTCA Systems was observed in S100Bwt treated in the tested concentration range (0.1-30 μM). Exposure to 5% DMSO as positive control decreased the cell index indicating neuronal cell death. **b)** The average number of primary, secondary, tertiary, and quaternary dendrites from at least 10 cells per condition was measured. No significant alterations were detected after treatment with S100B. **c)** Exemplary images show anti-MAP2 staining for the analysis of dendritic branching. **d)** The number of inhibitory synapses labeled by anti-Gephyrin staining is unaffected after treatment with S100B. **e)** The mRNA expression levels of *Shank2* and *Shank3* are not significantly altered after exposure to S100B or the S100Bmut (one-way ANOVA: *Shank2*: F_2,6_ = 0.469, *p* = 0.647; *Shank3*: F_2,6_ = 3.481, *p* = 0.099). **f)** Treatment of hippocampal cultures with 30 μM S100B and S100Bmut at DIV10 for 5 days. No reduction in immunofluorescence intensities of SHANK2 and SHANK3 immunoreactive puncta was seen (one way ANOVA: Shank2: F_2,27_ = 0.0886, p = 0.9155; Shank3: F_2,27_ = 0.8957, p = 0.4201).

**Supplementary Fig. S3:** **a-c)** Measurement of trace element levels in whole blood and brain tissue of mice using ICP-MS. **a)** The average whole-blood concentration of Fe, Cu, and Se is not significantly different between Ctrls- and S100B-injected pregnant mice (one-way ANOVA (n=3 per group)). **b)** The average whole blood concentration of Fe, Cu, and Se is not significantly different between pups from Ctrls (n=7) and S100B mice (n=4) (one-way ANOVA). c**)** The average concentration of Zn, Fe, Cu, and Se in whole brain tissue is not significantly different between saline-injected pregnant Ctrls and S100B-injected pregnant mice (one-way ANOVA (n=3 per group)).

**Supplementary Fig. S4: a)** In the light-dark test, % of time spent in the lighted zone of the shuttle box, **b,c)** in the marble burying test, the latency to bury the first marble **(b)** and the total number of buried marbles **(c)** were not significantly different between S100B and Ctrls mice.
