# Supplementary figures and images for: S100B dysregulation during brain development affects synaptic SHANK protein networks via alteration of zinc homeostasis
Source: Transl Psychiatry. 2021 Nov 5;11:562. doi: 10.1038/s41398-021-01694-z (PMC8571423; doi:10.1038/s41398-021-01694-z)

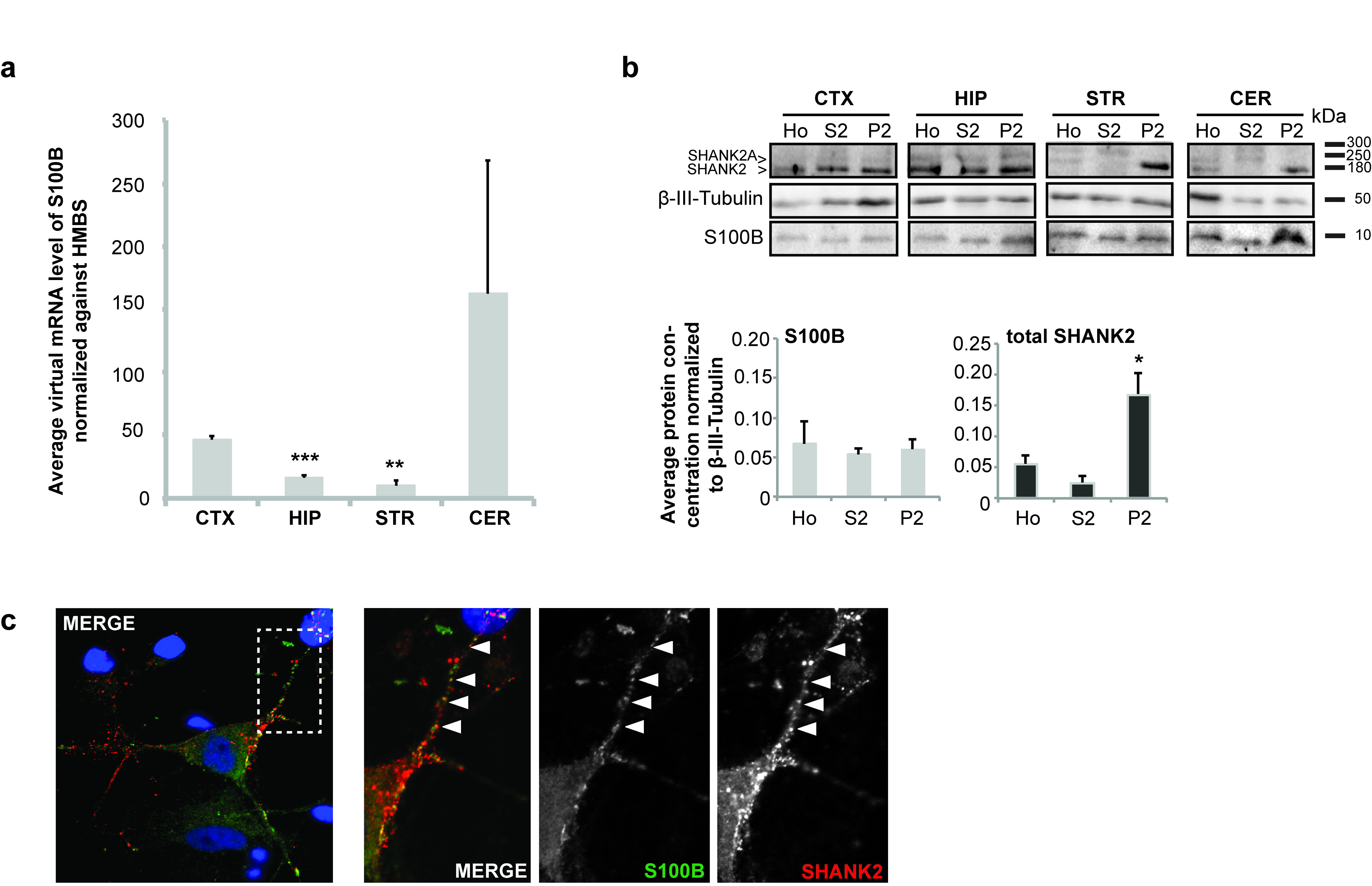

Supplement: Supplementary file 2 — Figure S1 [file 41398_2021_1694_MOESM2_ESM.tif]

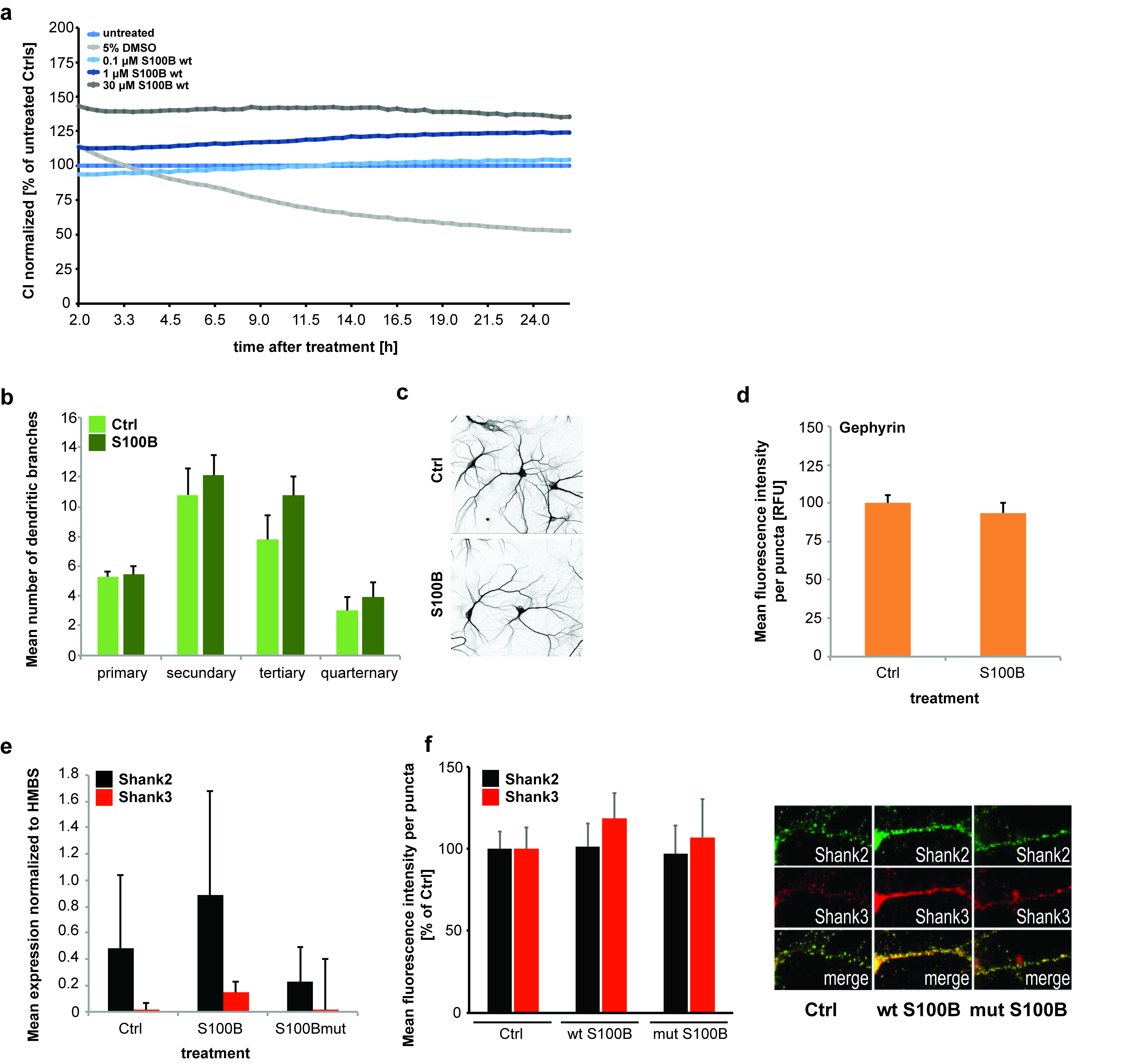

Supplement: Supplementary file 3 — Figure S2 [file 41398_2021_1694_MOESM3_ESM.tif]

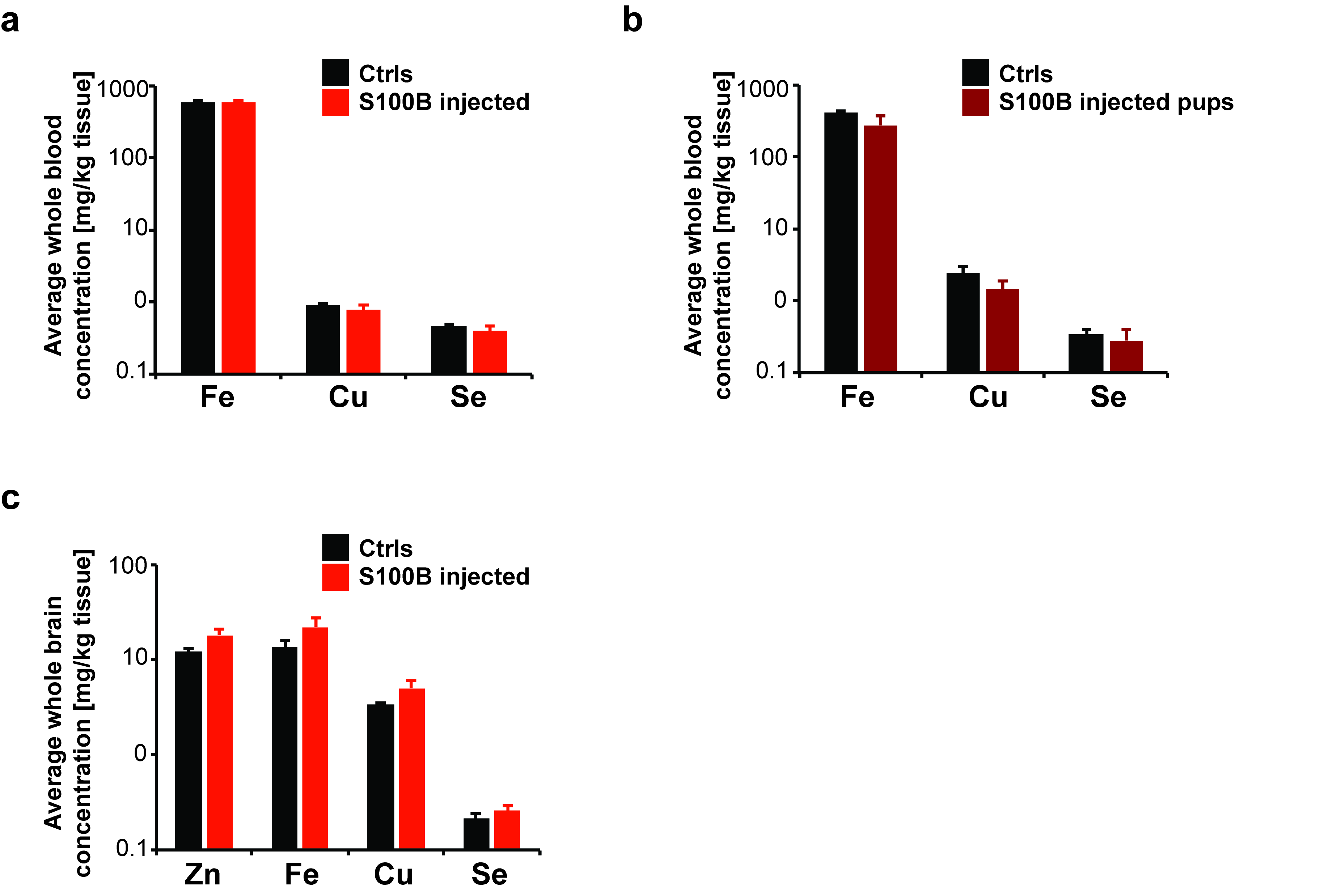

Supplement: Supplementary file 4 — Figure S3 [file 41398_2021_1694_MOESM4_ESM.tif]

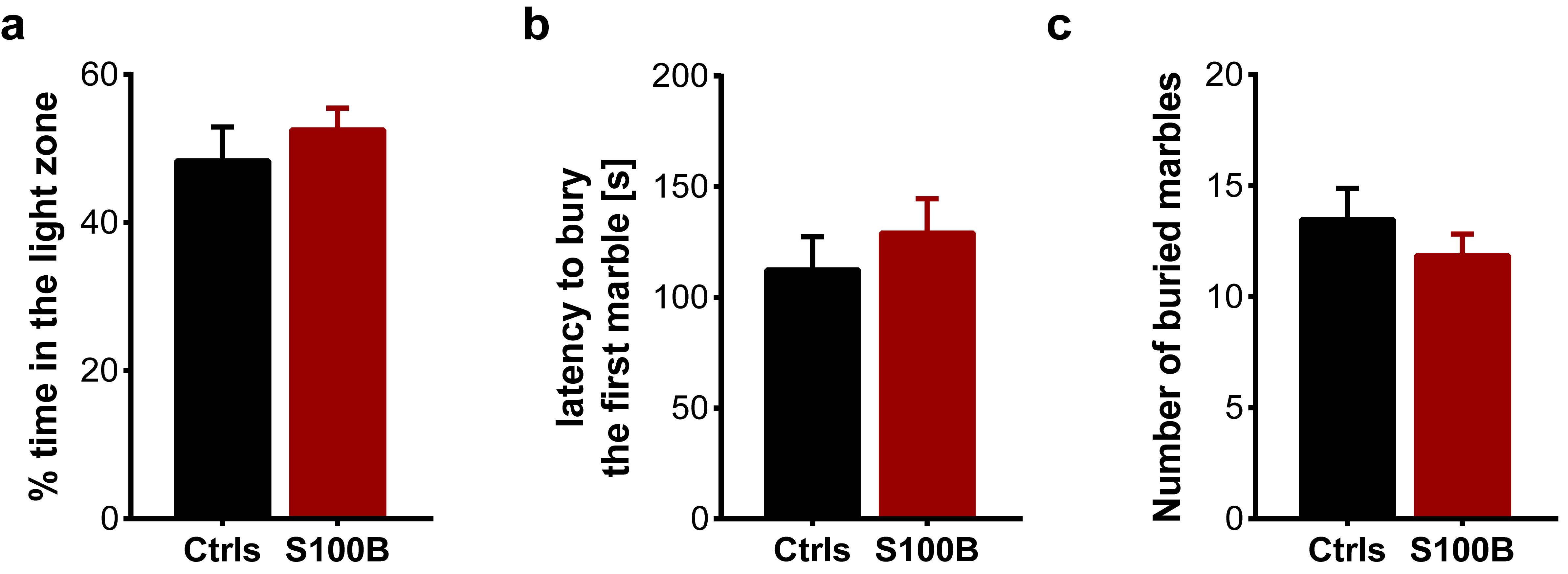

Supplement: Supplementary file 5 — Figure S4 [file 41398_2021_1694_MOESM5_ESM.tif]
